# Veterinary Medicine Students’ Perceptions of Hunting and Game Meat: A Cross-Sectional Survey at a Portuguese University

**DOI:** 10.3390/ani16081149

**Published:** 2026-04-09

**Authors:** Sara Marques, Ricardo J. Figueiredo, Alexandra Müller, Eduarda Gomes-Neves

**Affiliations:** 1 School of Medicine and Biomedical Sciences (ICBAS), University of Porto, Rua de Jorge Viterbo Ferreira 228, 4050-313 Porto, Portugal; samarques@icbas.up.pt (S.M.); up202206087@up.pt (R.J.F.); ammuller@icbas.up.pt (A.M.); 2 Interdisciplinary Centre of Marine and Environmental Research (CIIMAR)—Centre for Marine and Environmental Research (CIIMAR LA), University of Porto, Terminal de Cruzeiros do Porto de Leixões, Av. General Norton de Matos, S/N, 4450-208 Matosinhos, Portugal; 3 Centre for the Study of Animal Science (CECA)—Associate Laboratory for Animal and Veterinary Sciences (AL4AnimalS), Avenida da Universidade Técnica, 1300-477 Lisboa, Portugal

**Keywords:** game meat, hunting, veterinary education, wildlife health, one health, students’ choices, curriculum development

## Abstract

Background: Veterinarians are pivotal to wildlife health surveillance and game-meat safety, yet these topics receive limited emphasis in many veterinary curricula. Understanding students’ perceptions can inform targeted educational improvements. Methods: We conducted a cross-sectional, anonymous online survey among students enrolled in the Integrated Master’s in Veterinary Medicine at ICBAS-UP (Portugal). The questionnaire covered sociodemographic, meat and game-meat consumption, perceived appeal of working with game animals, and multi-select views on hunting, game-meat consumption and the veterinary role. We computed descriptive statistics and tested associations between categorical variables using Pearson’s Chi-square or Fisher’s exact tests (Monte Carlo correction when appropriate). Results: Of the 391 eligible students, 152 responded (39%). The majority (76%) associated hunting with veterinary inspection of game meat and research in epidemiology and emerging diseases, and many (72%) recognized as core roles monitoring the health of game animals and the contribution to public health and environmental sustainability. Significant associations included: prior game-meat consumption with finding game animals appealing/interest in learning more; year of enrolment with recognising hunting as an economic activity and acknowledging veterinary inspection and public health contributions; and perceiving game animals as appealing with associating hunting with population control (all *p* < 0.05; Cramer’s V indicating weak–moderate effects). Conclusions: Students show awareness of veterinary roles in game-animal health and meat inspection, but interest in working with game animals is low and knowledge gaps persist (e.g., inspection of game meat). Findings support curricular integration of wildlife health, game-meat inspection and One Health. Multicenter studies and evaluation of educational interventions are warranted.

## Simple Summary

Veterinarians play an important role in protecting the health of wild animals and ensuring that meat from hunted animals is safe for people to eat. To explore how future veterinarians view hunting and meat from wild animals, we asked all students enrolled in the Integrated Master’s of Veterinary Medicine of a veterinary school in Portugal to complete an online questionnaire. We intended to understand their experiences with consuming game meat, how they feel about working with game animals, and what ideas they associate with hunting. Furthermore, we investigated what roles they believe veterinarians should have in this area. About one-third of the students reported game meat consumption, while only a few felt that working with game animals was appealing, although many wished to learn more. Most students understood that veterinarians help protect public health by inspecting meat and studying diseases in wildlife. However, some important gaps in knowledge remained. These results show that adding more teaching about wildlife, hunting, and meat safety could better prepare future veterinarians and help society benefit from safer food and healthier ecosystems.

## 1. Introduction

Hunting and the control of game animal populations are crucial elements of wildlife management across Europe, playing significant roles in biodiversity conservation, ecosystem balance, and public health protection [1,2]. Hunting activities have been developed primarily for the procurement of nutrition, as well as being considered a sport. Effective management practices help mitigate conflicts arising from human–wildlife interactions, such as crop damage, vehicle collisions, and zoonotic disease transmission, thereby ensuring sustainable coexistence between wildlife populations and human activities [1].

Public perspectives on hunting, however, remain complex and multifaceted. Consumers increasingly express concerns about the ethical and ecological implications of hunting and the safety of game meat [2,3]. Ethical debates often revolve around animal welfare, sustainability, and humane treatment during hunting practices. Concurrently, meat safety concerns primarily focus on potential zoonotic pathogens, chemical contaminants, and residues from ammunition, particularly lead contamination, which pose significant health risks to consumers [2,4].

Veterinarians play a critical role in addressing these concerns through their involvement in wildlife disease surveillance, population control measures, and game meat inspection. By monitoring the health status of wild animal populations, veterinarians facilitate early detection and control of diseases such as chronic wasting disease, African swine fever, and bovine tuberculosis, thereby reducing risks to both animal and human health [5]. Veterinary expertise in meat inspection further ensures game meat safety by identifying pathological conditions, contamination, and ensuring compliance with hygiene regulations [6,7,8].

Given these responsibilities, it is essential to enhance veterinary training in the specific context of game animals and game meat inspection. Despite veterinary curricula covering wildlife health, epidemiology and meat safety in different courses, there is still some lack of consistency. Some differences were observed in relation to country-specific requirements (e.g., wild game meat hygiene in countries where hunting is common) [9,10]. This may need to be improved to ensure that graduates are not left inadequately prepared for the practical challenges they will face in the field [11]. Understanding veterinary medicine students’ perspectives regarding these topics is equally important. Assessing student awareness, attitudes, and perceived competence can highlight gaps in current educational frameworks, informing curriculum improvements that foster greater preparedness and professional awareness [12]. Enhanced awareness among veterinary students about wildlife conservation, ethical hunting practices, and public health aspects of game meat inspection can significantly contribute to their professional development, benefiting wildlife conservation efforts and public health protection.

The present study aims to assess veterinary students’ perceptions of hunting and game meat, to characterize their consumption habits and professional interest in these topics, and to explore factors associated with these perceptions across different stages of training.

## 2. Materials and Methods

### 2.1. Study Design and Population

This study was designed as a cross-sectional online survey targeting all students enrolled in the Integrated Master’s in Veterinary Medicine at ICBAS—University of Porto (Portugal). All academic years were eligible for participation. The Integrated Master’s program is accredited by both the European Committee of Veterinary Education (ECOVE) and the European Association of Establishments for Veterinary Education (EAEVE), ensuring compliance with recognized European veterinary education standards. This pilot study was conducted within the COST-Action Safety in the Game Meat Chain (CA22166).

### 2.2. Questionnaire

The questionnaire was developed according to established principles of online survey design, ensuring question simplicity, suitability for the target population, informed consent, and confidentiality [13]. The questionnaire contained an introductory section outlining the study’s objectives, emphasizing the voluntary nature of participation. The questionnaire consisted of ten closed-ended items grouped into two thematic blocks (Table 1). Block I (General Information) collected demographic and behavioral information (seven items), including year of enrolment, age, gender, dietary habits (meat and game meat consumption), being a hunter, and whether game animals were considered appealing to the veterinary profession. Block II (Opinions) examined students’ perceptions through three multi-select questions addressing (i) Associations they make with hunting, (ii) Associations they make with the consumption of game meat, and (iii) their understanding on the role of the veterinary medicine in relation to game animals and hunting. Participants were required to select at least one answer option for each item to ensure complete datasets. The questionnaire was reviewed and approved by the University of Porto Ethics Committee (protocol A52/2025).

### 2.3. Data Collection Procedures

Data were collected using a Google Forms survey, which was disseminated between 25 March and 2 April 2025. The survey link was distributed through multiple communication channels, including course classes, student email groups, and the representatives of each academic year. Reminder messages were sent during the data collection period to maximize participation. Responses from students not enrolled in the veterinary program, as well as incomplete surveys, were excluded from the analysis.

### 2.4. Statistical Analysis

Descriptive statistics were generated to summarize the distribution of responses, expressed as frequencies and percentages. Two main outcomes from Block I were analyzed for associations: (a) Ever consumed game meat (yes/no); (b) Perception of game animals as appealing to the veterinary profession (yes/no/would like to know more) with the remaining items of Block I (Table 2).

Subsequently, each variable from Block I was tested against every binary-coded response option from Block II (selected vs. not selected) (Table 3). Depending on expected cell counts, Pearson’s chi-square tests or Fisher’s exact tests (with Monte Carlo correction when required) were used to assess associations. Statistical significance was set at *p* < 0.05. Effect sizes were estimated using Cramer’s V or Phi coefficients, interpreted according to conventional thresholds (weak, moderate, strong). All analyses were performed using SPSS^®^ version 30 (IBM Corp., Armonk, NY, USA).

## 3. Results

### 3.1. Participation and Respondent Characteristics

A total of 152 students participated in the survey, corresponding to a response rate of 39% (152/391) (Table 1). All academic years were proportionally represented, with each cohort contributing between 11% and 20% of responses. The majority of respondents were female (79%), reflecting the gender distribution of the program. Most participants (76%) were 23 years of age or younger. Regarding dietary habits, 92% of the students reported that they consume meat. Game meat consumption was less common, with 38% indicating that they had eaten game meat at least once. When asked about the appeal of game animals to the veterinary profession, 59% considered them “not appealing,” while 33% expressed interest in learning more about this area. Only 8% of respondents found working with game species appealing.

### 3.2. Associations with Hunting and Game Meat

Students most frequently associated hunting with sport or leisure activities (84%). Other common associations included cultural traditions (51%), economic activity (44%), and population control of wildlife (42%). A smaller proportion (11%) linked hunting to land or natural resource management. Regarding game meat consumption, students commonly associated it with animals killed using firearms (70%), animals not raised in captivity (55%), and animals living in their natural habitat (45%). Notably, 39% of respondents associated game meat with the absence of official veterinary inspection. Fewer students associated game meat with environmental contaminants (15%) or with being organic and free from veterinary residues (11%). Students demonstrated relatively high awareness of the veterinary profession’s responsibilities concerning game animals. Specifically, 76% selected veterinary inspection of game meat as a professional role, and 76% recognized the role of veterinarians in epidemiological research on emerging diseases. Additionally, 72% identified both monitoring the health of game species and contributing to public health and environmental sustainability as appealing veterinary functions (Table 1).

### 3.3. Bivariate Associations

Several statistically significant associations emerged from the analysis (Table 2 and Table 3).

Game Meat Consumption and Interest in Game Animals (Table 2)

Students who had previously consumed game meat were significantly more likely to find game animals appealing (*n* = 8/13; 62%) or to express interest in learning more about them (*n* = 23/50; 54%) compared with students who had never consumed game meat (*n* = 26/89; 29%) (*p* = 0.024; Cramer’s V = 0.220, indicating a moderate effect).

Age and Perception of Game Animals (Table 2)

Perceiving game animals as appealing to the veterinary profession was associated with being older than 21 years (*n* = 10/96; 10%) compared to younger students (3/56; 5%) (*p* = 0.036). The effect size was weak, suggesting a modest relationship.

Year of Enrolment and Perceptions of Hunting, consumption of game meat and Veterinary Roles (Table 3)

A student’s year of enrolment was significantly associated with several perceptions. Students in earlier academic years were more likely to recognize hunting as an economic activity (*n* = 22/31; 71% of first-year students) (*p* = 0.007). More first-year students (*n* = 9/31; 29%) associated the consumption of game meat with organic meat without residues compared to students in later years (5th year students *n* = 1/27, 4%; internship students *n* = 2/16, 13%; *p* = 0.006). Year of enrolment was also associated with understanding the role of veterinarians in the inspection of game meat (4th year students *n* = 19/22, 86%; 5th year students *n* = 24/27, 89%; internship students *n* = 14/16, 88%; *p* < 0.001) and in public health and environmental sustainability (5th year students *n* = 24/27, 89%; internship students *n* = 12/16, 74%; *p* = 0.017). The effect sizes for these associations were moderate, indicating meaningful differences across stages of training.

Appeal of Game Animals to the Veterinary Profession and Associations with Hunting (Table 3)

Students who considered game animals appealing (80%) to the veterinary profession (*n* = 63) were significantly more likely to associate hunting with animal population control (*p* = 0.0005). This association also showed a moderate effect size.

## 4. Discussion

The questionnaire was structured according to domains commonly used in studies assessing attitudes toward wildlife, hunting, and food consumption. The first block captured demographic and behavioral variables known to influence perceptions in previous research, such as dietary practices and prior exposure to game meat. The second block included multi-choice items on associations with hunting, perceptions of game meat consumption, and awareness of veterinary roles, reflecting themes explored in earlier surveys of veterinary students and the general public. This structure ensured that the tool was appropriate for the target population—students with varying levels of exposure to wildlife topics—while enabling comparisons with analogous instruments used internationally. The questionnaire was designed to be concise, accessible, and suitable for classroom distribution, enhancing usability and response quality. As this was a pilot study using a questionnaire developed for this purpose, the findings should be interpreted in the context of a cross-sectional survey of a single institution. In this survey of veterinary students, most respondents recognized core professional functions related to game animals, particularly veterinary inspection of game meat and epidemiological research. However, relatively few found working with game species appealing.

Students who had consumed game meat were more likely to consider game animals appealing or to want to learn more. Later years of enrolment were associated with greater recognition of hunting as an economic activity and of veterinary roles in inspection and public health. Thematic Block I variables contributed important context to these perceptions. Prior game meat consumption showed the clearest behavioral association. This pattern aligns with European literature reporting that familiarity with hunting or game meat consumption often promotes more positive or engaged attitudes toward wildlife [2,14]. The relationship suggests that direct experiences, whether social or dietary, enhance students’ curiosity or perceived appeal of wildlife related topics. Also notably, nearly two in five students associated game meat with the absence of official veterinary inspection, indicating a specific knowledge gap regarding the European regulatory framework for wild game placed on the market. This misconception may be related to the fact that it is common in Portugal as in other countries, that most of the meat from the hunting activity is consumed directly by the hunters without the placement on the market [15,16,17]. These findings align with European literature showing that familiarity with hunting and game meat is associated with more favorable attitudes, while food safety concerns remain prominent barriers to consumption [2,18,19]. Within the EU, wild game intended for placing on the market is subject to the same hygiene and official control architecture governing other products of animal origin, Reg. (EC) 853/2004, Reg. (EU) 2017/625, and Implementing Reg. (EU) 2019/627, so the belief that marketed game meat is categorically “without official veterinary inspection” is inaccurate [6,7,8]. Strengthening teaching on the regulatory pathways for harvested game—distinguishing between direct consumption and market placement—may therefore be particularly beneficial.

Age and year of enrolment also played important roles. Older students were modestly more likely to find game species appealing to the profession, a pattern that may reflect maturing interests, greater exposure to diverse veterinary topics throughout the curriculum, or broader life experience. Although the effect size was weak, this association reinforces the idea that students’ perspectives may evolve as they progress through their studies and accumulate more academic and professional context. Although associations showed weak to moderate effect sizes, they remain relevant in light of evolving EU food-safety obligations and Day-1 competencies, which require veterinarians to understand wildlife-associated risks and regulatory frameworks. Year of enrolment was associated with several perceptions of hunting, game meat consumption, and veterinary roles. Students in earlier years were more likely to associate hunting with economic activity and to view game meat as “organic meat without residues,” whereas students further along in their training showed stronger recognition of veterinary inspection, public health contributions, and environmental sustainability. These findings suggest that the curriculum progressively strengthens students’ understanding of food safety, hygiene, and public health frameworks, and that early-year perceptions may be influenced more by public or cultural narratives than by technical knowledge [20,21].

Regarding gender, although no statistically significant associations were detected, the strong female predominance in the sample reflects the wider demographic profile of veterinary education. Gender has been shown in previous studies to influence attitudes toward hunting and animal use ethics, and researchers generally recommend acknowledging these dynamics when interpreting survey patterns [2]. In this study, gender does not appear to drive major attitudinal differences, but the skewed distribution reinforces the need to engage a diverse student population when teaching wildlife and hunting related topics.

The variable “eating meat in general” showed no association with perceptions of game animals or hunting. This finding suggests that students’ views on hunting and game meat are not simply extensions of broader dietary habits. Rather, perceptions may be more strongly influenced by cultural exposure, personal experiences, risk perceptions, and curricular content.

Regarding ethical issues and public acceptance, students most often associate hunting with sport/leisure, followed by cultural tradition, economic activity, and population control. This pattern mirrors European surveys showing higher acceptance when hunting is linked to meat provision and wildlife population management, and lower acceptance when framed solely as “sport.” Direct experience, through social networks or consumption of game meat, predicts more positive attitudes, consistent with our observation that prior game meat consumption correlates with greater interest in game species [2,18,19]. In parallel, the literature highlights ethical/sustainability debates, consumer awareness gaps, and perceived safety risks as barriers, areas where veterinary professionals can contribute through evidence-based risk communication and supply chain assurance [2].

In terms of food safety concerns, the frequent association of game meat with a lack of inspection likely coexists with concerns about chemical and physical hazards, particularly lead from ammunition. Reviews and imaging studies show that lead gunshot and some bullets fragment extensively, depositing micro and millimetre-scale particles that are difficult to remove during preparation; frequent consumers and vulnerable groups are therefore at risk of elevated dietary exposure [22,23]. Transition to non-lead ammunition demonstrably reduces lead in meat (e.g., Denmark), and EU level risk management proposals under REACH have advanced through ECHA’s scientific committees and targeted consultations [24,25]. Training that covers (i) hazard identification (lead, zoonoses), (ii) field to fork control points (e.g., trimming around the wound channel, hygienic dressing, offal management), and (iii) communication for hunters and consumers would directly address students’ uncertainties.

Students’ recognition of veterinary contributions to epidemiology, surveillance, and public health is encouraging. The One Health literature identifies wildlife as critical in the emergence and maintenance of shared infections; establishing fit for purpose wildlife disease surveillance is repeatedly recommended as a prerequisite for intervention [5,26]. Reviews at the wildlife–livestock–human interface emphasize integrated strategies, preventive actions, vector control, host population management, vaccination where feasible, and stress that surveillance (disease and population wise) must underpin decisions to intervene [26].

Illustrative examples from Europe include African swine fever (ASF), as it exemplifies these principles: EFSA’s reporting and guidance highlight the importance of passive surveillance in wild boar, carcass removal, biosecurity, and coordinated action across hunters, veterinarians, and authorities [27,28,29]. Embedding case-based teaching on ASF (and other exemplars such as bovine tuberculosis) can ground students’ perceptions in current surveillance and control realities. For bovine tuberculosis, an enduring multi-host problem in several regions, recent reviews and international perspectives reaffirm the complexity of cross-species transmission and the need for surveillance strategies and tools tailored to local wildlife communities [30,31].

Two immediate educational implications emerge. First, a Wildlife & Game Meat Public Health module (spiral across years) could cover: EU law for wild game (Reg. 853/2004; Reg. 2017/625; Impl. Reg. 2019/627), inspection flow from field to game handling establishments, hazard control (lead fragment mitigation, hygienic dressing, zoonoses), and communication for risk perception and ethics, directly addressing the misbelief regarding inspection [6,7,8]. Second, align the curriculum with WOAH Day 1 competencies and One Health expectations, food hygiene/meat inspection, epidemiology, zoonoses, transboundary diseases, and communication, using structured methodologies to diagnose gaps and implement iterative improvements with stakeholder participation [32,33].

This study has several limitations. First, it represents the experience of a single European veterinary school, which restricts the generalizability of the findings to other educational, cultural, and regulatory contexts. Similar constraints have been noted in previous research on hunting attitudes and game-meat perceptions, where national context and local familiarity strongly influence results. Second, the modest response rate and use of closed-ended survey items limit the depth of interpretation, as complex attitudes toward hunting, wildlife ethics, and food safety often require qualitative exploration to fully capture student reasoning. Future work should therefore expand this survey to multiple veterinary universities across Europe, enabling comparison across different curricula, wildlife-health exposure, and national regulatory environments. Multi-country studies would also align with calls in the literature to better understand regional variation in social acceptance of hunting, game-meat consumption, and wildlife-related veterinary roles in Europe. Such an expanded approach would strengthen the evidence base for harmonized European veterinary-curriculum development in wildlife health, game-meat inspection, and One Health.

## 5. Conclusions

Veterinary students show meaningful awareness of surveillance, inspection, and public-health roles in relation to hunting and game meat, but interest in specializing in game species remains low, and specific knowledge gaps persist, especially around official inspection of marketed game meat and food-safety hazards such as lead. Given the One Health significance of wildlife management and game-meat safety in Europe, targeted curricular integration, linked to EU law and WOAH Day-1 competencies, can translate students’ awareness into professional readiness to protect consumers, wildlife, and livestock alike [4,5,17].

## Figures and Tables

**Table 1:** Descriptive characteristics of responses provided by veterinary medicine students (N = 152) to all items of the questionnaire, organized by Thematic Block I (General Information) and Thematic Block II (Opinions). Frequencies and percentages are presented for each response option. Multiple-choice questions may exceed 100% due to more than one response allowed.

Questionnaire	Question	Response Option	Number (%)
Thematic Block I	Year of enrolment?	1st	31 (20%)
		2nd	30 (20%)
		3rd	26 (17%)
		4th	22 (14%)
		5th	27 (18%)
		Internship	16 (11%)
	Age?	18–20 years	56 (37%)
		21–23 years	60 (39%)
		>23 years	36 (24%)
	Gender?	Female	120 (79%)
		Male	29 (19%)
		No answer	3 (2%)
	Are you a hunter?	Yes	1 (1%)
		No	151 (99%)
	Do you eat meat in general?	Yes	140 (92%)
		No	12 (8%)
	Have you ever eaten game meat?	Yes	57 (38%)
		No	95 (62%)
	In the field of veterinary medicine, do you consider game animals appealing to the veterinary profession?	Yes	13 (8%)
		No	89 (59%)
		Would like to know more	50 (33%)
Thematic Block II *	What do you associate with hunting?	Sports and leisure activities	127 (84%)
		Cultural and traditional activity	78 (51%)
		Economic activity	67 (44%)
		Animal population control	64 (42%)
		Land use planning and natural resources management	16 (11%)
	What do you associate with the consumption of game meat?	Animals slaughtered using a firearm	106 (70%)
		Animals not raised in captivity	83 (55%)
		Animals in their natural habitat	69 (45%)
		Without official veterinary inspection	60 (39%)
		With residues of environmental contaminants	23 (15%)
		Organic meat, without residues of veterinary products	17 (11%)
	What is your view on the role of veterinary medicine in relation to game animals and hunting?	Veterinary inspection of game meat	115 (76%)
		Research in epidemiology and emerging diseases	116 (76%)
		Monitoring the health of game animals	110 (72%)
		Contribution to public health and environmental sustainability	110 (72%)

* The sum exceeds 100% due to multiple choices. Source: Data derived from the present study.

**Table 2:** Associations between two key variables from Thematic Block I—(a) having ever consumed game meat and (b) considering game animals appealing to the veterinary profession—and the remaining items of Thematic Block I. Statistically significant results (*p* < 0.05) are shown in bold. Effect sizes are reported using Cramer’s V.

	Have You Ever Eaten Game Meat?		Do You Consider Game Animals Appealing to the Veterinary Profession?	
	Chi Square *p* Value	Strength of Association Cramer’s V	Chi Square *p* Value	Strength of Association Cramer’s V
Year of enrolment?	0.600 ^a^	-	0.369 ^b^	-
Age?	0.227 ^a^	-	**0.036 ^b^**	0.183 (weak 0.1–0.2)
Are you a hunter?	0.375 ^c^	-	0.086 ^c^	-
Do you eat meat in general?	0.536 ^c^	-	0.899 ^c^	-
Have you ever eaten game meat?	n.a.		n.a.	
Do you consider game animals appealing to the veterinary profession?	**0.024 ^b^**	0.220 (moderate 0.2–0.4)	n.a.	

^a^ Pearson Chi-Square ^b^ Pearson Chi-square with Monte Carlo correction; ^c^ Fishers exact test. Source: Data derived from the present study. n.a.—not available.

**Table 3:** Statistically significant associations between variables from Thematic Block I and individual response options of Thematic Block II. Response options in Thematic Block II were converted to binary variables (selected vs. not selected). Associations were tested using Pearson’s chi-square with Monte Carlo correction when required. Effect sizes are reported using Cramer’s V.

		Thematic Block II			
Thematic Block I	**Question**	**Question**	**Response option**	**Chi square** ***p* value**	**Strength of association** **Cramer’s V**
	Year of enrolment?	What do you associate with hunting?	Economic activity (Yes, No)	0.007 ^b^	0.319 (moderate 0.2–0.4)
		What do you associate with the consumption of game meat?	Organic meat without residues (Yes, No)	0.006 ^b^	0.323 (moderate 0.2–0.4)
		What is your view on the role of veterinary medicine in relation to game animals and hunting?	Veterinary inspection of game meat	<0.001 ^b^	0.381 (moderate 0.2–0.4)
			Contribution to public health and environmental sustainability	0.017 ^b^	0.301 (moderate 0.2–0.4)
	In the field of veterinary medicine, do you consider game animals appealing to the veterinary profession?	What do you associate with hunting?	Animal population control (Yes, No)	0.0005 ^a^	0.263 (moderate 0.2–0.4)

^a^ Pearson Chi-Square ^b^ Pearson Chi-square with Monte Carlo correction. Source: Data derived from the present study.

## Data Availability

The datasets generated and analyzed during the current study are not publicly available due to confidentiality and privacy considerations related to participant responses but are available from the corresponding author upon reasonable request.
